# Ethyl 4-acetyl-5-anilino-3-methyl­thio­phene-2-carboxyl­ate

**DOI:** 10.1107/S160053681301547X

**Published:** 2013-06-08

**Authors:** Yahia Nasser Mabkhot, Fatima Alatibi, Assem Barakat, M. Iqbal Choudhary, Sammer Yousuf

**Affiliations:** a Department of Chemistry, College of Science, King Saud University, PO Box 2455, Riyadh 11451, Saudi Arabia; b Department of Chemistry, Faculty of Science, Alexandria University, PO Box 426, Ibrahimia 21321 Alexandria, Egypt; c H.E.J. Research Institute of Chemistry, International Center for Chemical and Biological Sciences, University of Karachi, Karachi 75270, Pakistan

## Abstract

In the title compound, C_16_H_17_NO_3_S, a thio­phene derivative with amino phenyl, acetyl, methyl and ethyl carboxyl susbtituents attached to a central thio­phene ring, the phenyl and thio­phene rings form a dihedral angle of 36.92 (9) Å. The mol­ecular conformation is stabilized by an intra­molecular N—H⋯O hydrogen bond, which forms an *S*(6) ring motif.

## Related literature

For the biological activity of thio­phene derivatives, see: Mishra *et al.* (2011 ▶); Mabkhot *et al.* (2013*b* ▶). For the synthesis of fused heterocyclic compounds, see: Sommen *et al.* (2003 ▶). For crystal data for related thio­phene compounds, see: Mabkhot *et al.* (2013*a* ▶,*b* ▶); Buehrdel *et al.* (2007 ▶).
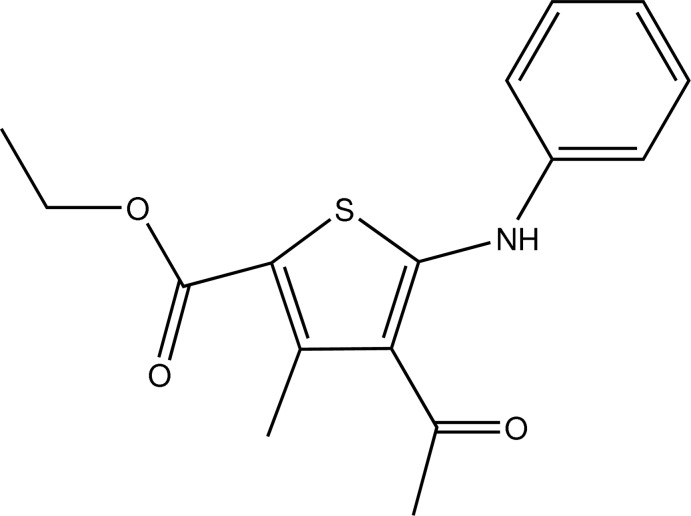


## Experimental

### 

#### Crystal data

C_16_H_17_NO_3_S
*M* *_r_* = 303.37
Triclinic, 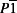

*a* = 7.9443 (6) Å
*b* = 9.5038 (7) Å
*c* = 11.8706 (9) Å
α = 66.759 (2)°
β = 89.754 (2)°
γ = 66.785 (2)°
*V* = 744.60 (10) Å^3^
*Z* = 2
Mo *K*α radiation
μ = 0.23 mm^−1^
*T* = 273 K
0.45 × 0.42 × 0.23 mm

#### Data collection

Bruker SMART APEX CCD area-detector diffractometer
Absorption correction: multi-scan (*SADABS*; Bruker, 2000 ▶) *T*_min_ = 0.905, *T*_max_ = 0.950
10410 measured reflections
3699 independent reflections
2848 reflections with *I* > 2σ(*I*)
*R*_int_ = 0.028

#### Refinement

*R*[*F* ^2^ > 2σ(*F* ^2^)] = 0.048
*wR*(*F* ^2^) = 0.126
*S* = 1.05
3699 reflections
194 parameters
H atoms treated by a mixture of independent and constrained refinement
Δρ_max_ = 0.30 e Å^−3^
Δρ_min_ = −0.25 e Å^−3^

### 

Data collection: *SMART* (Bruker, 2000 ▶); cell refinement: *SAINT* (Bruker, 2000 ▶); data reduction: *SAINT*; program(s) used to solve structure: *SHELXS97* (Sheldrick, 2008 ▶); program(s) used to refine structure: *SHELXL97* (Sheldrick, 2008 ▶); molecular graphics: *SHELXTL*; software used to prepare material for publication: *SHELXTL*, *PARST* (Nardelli, 1995 ▶) and *PLATON* (Spek, 2009 ▶).

## Supplementary Material

Crystal structure: contains datablock(s) global, I. DOI: 10.1107/S160053681301547X/rz5071sup1.cif

Structure factors: contains datablock(s) I. DOI: 10.1107/S160053681301547X/rz5071Isup2.hkl

Supplementary material file. DOI: 10.1107/S160053681301547X/rz5071Isup3.cml

Additional supplementary materials:  crystallographic information; 3D view; checkCIF report

## Figures and Tables

**Table 1:** Hydrogen-bond geometry (Å, °)

*D*—H⋯*A*	*D*—H	H⋯*A*	*D*⋯*A*	*D*—H⋯*A*
N1—H1*A*⋯O1	0.82 (3)	1.93 (3)	2.607 (3)	140 (2)

**Fig. 1.**
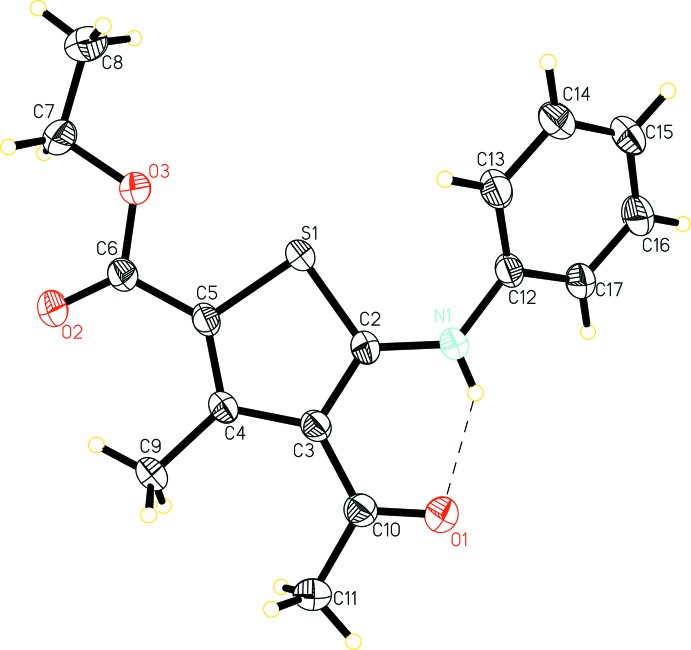
The molecular structure of the title compound with displacement ellipsoids drawn at the 30% probability level. The dashed line indicates the intramolecular hydrogen bond.

**Fig. 2.**
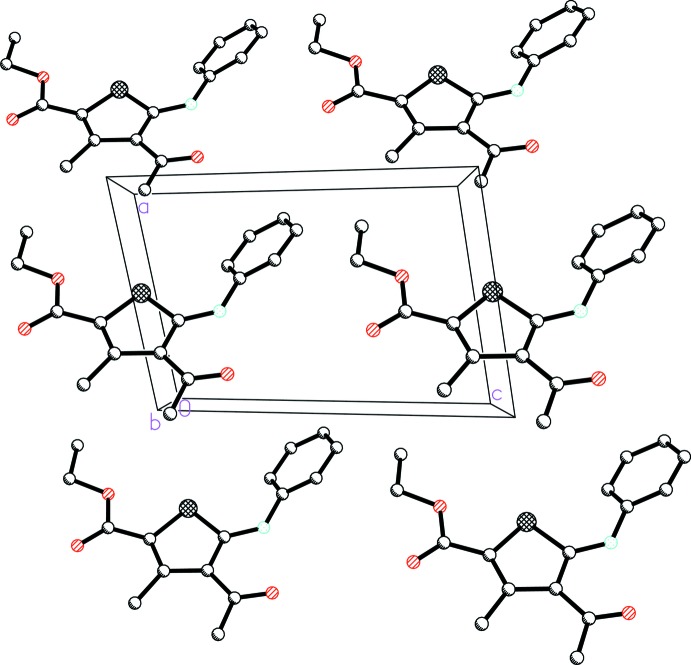
Crystal packing of the title compound viewed down the b axis. Hydrogen atoms are ommited for clarity.

## supplementary crystallographic information

### Comment

Sulfur containing heterocyclic compounds are well known for their diverse range of biological activities (Mabkhot *et al.*, 2013*b*; Mishra *et al.* 2011). The title compound was synthesized in continuation of our research towards the synthesis of biologically active compounds with fused heterocyclic systems (Mabkhot *et al.*, 2013*b*).

The structure of the title compound (Fig. 1) is composed of a planar thiophene (S1/C2–C5) ring with amino phenyl (N1/C12–C17), acetyl (O1/C10–C11), methyl (C8) and ethyl carboxylate (O2–O3/N2/C6–C7) susbtituents attached to atoms C1, C2,C3 and C4, respectively, of the thiophene ring. The dihedral angle between the planes of thiophene and amino phenyl ring (N1/C11–C16) is 36.92 (9)°. The bond lengths and angles are similar to those of structurally related compounds (Mabkhot *et al.*, 2013*a*,*b*; Buehrdel *et al.*, 2007). The molecular conformation is stabilized by an N1—H1A···O1 intramolecular hydrogen bond (Table 1) to form a *S*6 graph set ring motif. In the crystal packing (Fig. 2), no π···π or C—H···π interactions are observed between adjacent molecules.

### Experimental

The title compound was synthesized by the procedure described in the literature (Sommen *et al.*, 2003). The compound was crystallized by using a mixture of dimethyl formamide and dichloromethane 1:1 *v*/*v* at room temperature. M. p.: 399 K. Spectral Data: IR (KBr, cm^-1^): 1680, 1700, 2990 cm^-1^; ^1^H-NMR (400 MHz, CDCl_3_): δ 1.34 (t, 3H, *J* = 7.1 Hz), 2.58 (s, 3H), 2.82 (s, 3H), 4.28 (q, 2H, *J* = 7.1 Hz), 7.35–7.42 (m, 5H), 12.1 (s, 1H); ^13^C-NMR (100 MHz, CDCl_3_): δ 14.3, 16.5, 31.3, 60.5, 108.9, 119.2, 120.5, 124.7 (2 C), 129.5 (2 C), 139.6, 145.8, 162.7, 163.4, 195.7; Anal. calcd for C_16_H_17_NO_3_S: C 63.34; H 5.65; N 4.62. Found: C 63.47; H 5.46; N 4.61.

### Refinement

H atoms on methyl, methylene and methine were positioned geometrically with C—H = 0.96 Å, 0.97 Å and 0.93 Å respectively, and constrained to ride on their parent atoms with *U*_iso_(H)= 1.2*U*_eq_ (CH_2_, CH) and 1.5*U*_eq_(CH_3_). The H atoms on the nitrogen atom was located in a difference Fourier map and refined isotropically (N–H= 0.82 (3) Å).

### Crystal data

**Table:**

C_16_H_17_NO_3_S	*Z* = 2
*M**_r_* = 303.37	*F*(000) = 320
Triclinic, *P*1	*D*_x_ = 1.353 Mg m^−^^3^
Hall symbol: -P 1	Mo *K*α radiation, λ = 0.71073 Å
*a* = 7.9443 (6) Å	Cell parameters from 2619 reflections
*b* = 9.5038 (7) Å	θ = 2.5–25.8°
*c* = 11.8706 (9) Å	µ = 0.23 mm^−^^1^
α = 66.759 (2)°	*T* = 273 K
β = 89.754 (2)°	Block, yellow
γ = 66.785 (2)°	0.45 × 0.42 × 0.23 mm
*V* = 744.60 (10) Å^3^	

### Data collection

**Table:**

Bruker SMART APEX CCD area-detector diffractometer	3699 independent reflections
Radiation source: fine-focus sealed tube	2848 reflections with *I* > 2σ(*I*)
Graphite monochromator	*R*_int_ = 0.028
ω scan	θ_max_ = 28.3°, θ_min_ = 1.9°
Absorption correction: multi-scan (*SADABS*; Bruker, 2000)	*h* = −10→10
*T*_min_ = 0.905, *T*_max_ = 0.950	*k* = −12→12
10410 measured reflections	*l* = −15→15

### Refinement

**Table:**

Refinement on *F*^2^	Primary atom site location: structure-invariant direct methods
Least-squares matrix: full	Secondary atom site location: difference Fourier map
*R*[*F*^2^ > 2σ(*F*^2^)] = 0.048	Hydrogen site location: inferred from neighbouring sites
*wR*(*F*^2^) = 0.126	H atoms treated by a mixture of independent and constrained refinement
*S* = 1.05	*w* = 1/[σ^2^(*F*_o_^2^) + (0.0618*P*)^2^ + 0.0954*P*] where *P* = (*F*_o_^2^ + 2*F*_c_^2^)/3
3699 reflections	(Δ/σ)_max_ < 0.001
194 parameters	Δρ_max_ = 0.30 e Å^−^^3^
0 restraints	Δρ_min_ = −0.25 e Å^−^^3^

### Special details

**Table:**

Geometry. All e.s.d.'s (except the e.s.d. in the dihedral angle between two l.s. planes) are estimated using the full covariance matrix. The cell e.s.d.'s are taken into account individually in the estimation of e.s.d.'s in distances, angles and torsion angles; correlations between e.s.d.'s in cell parameters are only used when they are defined by crystal symmetry. An approximate (isotropic) treatment of cell e.s.d.'s is used for estimating e.s.d.'s involving l.s. planes.
Refinement. Refinement of *F*^2^ against ALL reflections. The weighted *R*-factor *wR* and goodness of fit *S* are based on *F*^2^, conventional *R*-factors *R* are based on *F*, with *F* set to zero for negative *F*^2^. The threshold expression of *F*^2^ > σ(*F*^2^) is used only for calculating *R*-factors(gt) *etc*. and is not relevant to the choice of reflections for refinement. *R*-factors based on *F*^2^ are statistically about twice as large as those based on *F*, and *R*- factors based on ALL data will be even larger.

### Fractional atomic coordinates and isotropic or equivalent isotropic displacement parameters (Å^2^)

**Table:**

	*x*	*y*	*z*	*U*_iso_*/*U*_eq_	
S1	0.48333 (6)	0.26835 (5)	−0.00730 (4)	0.04325 (15)	
O1	0.8347 (2)	0.01329 (18)	−0.22852 (13)	0.0685 (4)	
O2	0.6413 (2)	−0.06433 (17)	0.34080 (12)	0.0704 (5)	
O3	0.41944 (19)	0.19842 (16)	0.23323 (11)	0.0546 (3)	
N1	0.5630 (2)	0.2832 (2)	−0.23190 (14)	0.0483 (4)	
C2	0.6046 (2)	0.1930 (2)	−0.10687 (15)	0.0404 (4)	
C3	0.7453 (2)	0.0270 (2)	−0.04358 (15)	0.0407 (4)	
C4	0.7482 (2)	−0.0370 (2)	0.08869 (15)	0.0394 (4)	
C5	0.6149 (2)	0.0774 (2)	0.12066 (15)	0.0419 (4)	
C6	0.5653 (3)	0.0580 (2)	0.24344 (16)	0.0475 (4)	
C7	0.3506 (3)	0.1937 (3)	0.34727 (19)	0.0634 (6)	
H7A	0.4503	0.1639	0.4111	0.076*	
H7B	0.2996	0.1109	0.3776	0.076*	
C8	0.2028 (3)	0.3670 (3)	0.3158 (2)	0.0728 (6)	
H8A	0.1526	0.3702	0.3889	0.109*	
H8B	0.1054	0.3948	0.2524	0.109*	
H8C	0.2553	0.4475	0.2858	0.109*	
C9	0.8800 (3)	−0.2083 (2)	0.18489 (17)	0.0539 (5)	
H9A	0.8528	−0.2197	0.2661	0.081*	
H9B	1.0055	−0.2205	0.1820	0.081*	
H9C	0.8659	−0.2944	0.1682	0.081*	
C10	0.8559 (2)	−0.0600 (2)	−0.11438 (17)	0.0466 (4)	
C11	0.9989 (3)	−0.2421 (3)	−0.0534 (2)	0.0605 (5)	
H11A	1.0558	−0.2749	−0.1159	0.091*	
H11B	0.9394	−0.3133	−0.0102	0.091*	
H11C	1.0925	−0.2538	0.0049	0.091*	
C12	0.4166 (2)	0.4428 (2)	−0.30492 (15)	0.0435 (4)	
C13	0.3570 (3)	0.5761 (2)	−0.27179 (18)	0.0536 (5)	
H13A	0.4097	0.5614	−0.1958	0.064*	
C14	0.2190 (3)	0.7316 (2)	−0.3516 (2)	0.0640 (6)	
H14A	0.1781	0.8210	−0.3286	0.077*	
C15	0.1418 (3)	0.7551 (3)	−0.4643 (2)	0.0665 (6)	
H15A	0.0501	0.8603	−0.5181	0.080*	
C16	0.2008 (3)	0.6224 (3)	−0.49714 (19)	0.0653 (6)	
H16A	0.1484	0.6380	−0.5735	0.078*	
C17	0.3366 (3)	0.4667 (2)	−0.41818 (17)	0.0540 (5)	
H17A	0.3750	0.3772	−0.4409	0.065*	
H1A	0.627 (3)	0.228 (3)	−0.267 (2)	0.058 (6)*	

### Atomic displacement parameters (Å^2^)

**Table:**

	*U*^11^	*U*^22^	*U*^33^	*U*^12^	*U*^13^	*U*^23^
S1	0.0447 (3)	0.0347 (2)	0.0357 (2)	−0.00843 (18)	0.00177 (18)	−0.00958 (17)
O1	0.0810 (10)	0.0576 (9)	0.0452 (8)	−0.0093 (8)	0.0116 (7)	−0.0217 (7)
O2	0.0924 (11)	0.0462 (8)	0.0366 (7)	−0.0056 (7)	0.0057 (7)	−0.0072 (6)
O3	0.0653 (8)	0.0428 (7)	0.0390 (7)	−0.0107 (6)	0.0109 (6)	−0.0139 (6)
N1	0.0532 (9)	0.0401 (8)	0.0330 (8)	−0.0071 (7)	0.0017 (7)	−0.0105 (6)
C2	0.0419 (9)	0.0394 (9)	0.0344 (8)	−0.0161 (7)	0.0013 (7)	−0.0119 (7)
C3	0.0382 (9)	0.0379 (8)	0.0387 (9)	−0.0121 (7)	0.0011 (7)	−0.0136 (7)
C4	0.0379 (8)	0.0348 (8)	0.0369 (8)	−0.0121 (7)	−0.0019 (7)	−0.0105 (7)
C5	0.0459 (9)	0.0357 (8)	0.0331 (8)	−0.0132 (7)	−0.0013 (7)	−0.0084 (7)
C6	0.0573 (11)	0.0370 (9)	0.0381 (9)	−0.0144 (8)	0.0025 (8)	−0.0121 (7)
C7	0.0785 (15)	0.0569 (12)	0.0454 (11)	−0.0198 (11)	0.0207 (10)	−0.0223 (10)
C8	0.0731 (15)	0.0667 (14)	0.0759 (16)	−0.0192 (12)	0.0235 (13)	−0.0386 (13)
C9	0.0515 (11)	0.0418 (10)	0.0426 (10)	−0.0044 (8)	−0.0029 (8)	−0.0090 (8)
C10	0.0447 (10)	0.0452 (10)	0.0461 (10)	−0.0156 (8)	0.0045 (8)	−0.0194 (8)
C11	0.0581 (12)	0.0510 (11)	0.0601 (13)	−0.0085 (9)	0.0104 (10)	−0.0268 (10)
C12	0.0440 (9)	0.0390 (9)	0.0351 (9)	−0.0153 (7)	0.0041 (7)	−0.0065 (7)
C13	0.0664 (13)	0.0430 (10)	0.0415 (10)	−0.0210 (9)	−0.0020 (9)	−0.0107 (8)
C14	0.0776 (15)	0.0396 (10)	0.0563 (13)	−0.0154 (10)	0.0040 (11)	−0.0126 (9)
C15	0.0637 (13)	0.0466 (11)	0.0528 (12)	−0.0064 (10)	−0.0046 (10)	−0.0034 (9)
C16	0.0684 (14)	0.0613 (13)	0.0417 (11)	−0.0148 (11)	−0.0086 (10)	−0.0112 (10)
C17	0.0619 (12)	0.0493 (10)	0.0368 (10)	−0.0146 (9)	0.0011 (8)	−0.0140 (8)

### Geometric parameters (Å, º)

**Table:**

S1—C2	1.7182 (18)	C8—H8C	0.9600
S1—C5	1.7422 (16)	C9—H9A	0.9600
O1—C10	1.230 (2)	C9—H9B	0.9600
O2—C6	1.202 (2)	C9—H9C	0.9600
O3—C6	1.339 (2)	C10—C11	1.509 (3)
O3—C7	1.449 (2)	C11—H11A	0.9600
N1—C2	1.350 (2)	C11—H11B	0.9600
N1—C12	1.409 (2)	C11—H11C	0.9600
N1—H1A	0.82 (2)	C12—C13	1.379 (3)
C2—C3	1.409 (2)	C12—C17	1.384 (3)
C3—C4	1.439 (2)	C13—C14	1.382 (3)
C3—C10	1.460 (2)	C13—H13A	0.9300
C4—C5	1.365 (2)	C14—C15	1.371 (3)
C4—C9	1.500 (2)	C14—H14A	0.9300
C5—C6	1.468 (2)	C15—C16	1.372 (3)
C7—C8	1.491 (3)	C15—H15A	0.9300
C7—H7A	0.9700	C16—C17	1.374 (3)
C7—H7B	0.9700	C16—H16A	0.9300
C8—H8A	0.9600	C17—H17A	0.9300
C8—H8B	0.9600		
			
C2—S1—C5	91.06 (8)	C4—C9—H9B	109.5
C6—O3—C7	116.03 (14)	H9A—C9—H9B	109.5
C2—N1—C12	129.39 (17)	C4—C9—H9C	109.5
C2—N1—H1A	111.3 (15)	H9A—C9—H9C	109.5
C12—N1—H1A	118.8 (15)	H9B—C9—H9C	109.5
N1—C2—C3	124.72 (16)	O1—C10—C3	120.70 (17)
N1—C2—S1	122.77 (13)	O1—C10—C11	116.71 (17)
C3—C2—S1	112.47 (12)	C3—C10—C11	122.59 (17)
C2—C3—C4	111.16 (15)	C10—C11—H11A	109.5
C2—C3—C10	119.78 (15)	C10—C11—H11B	109.5
C4—C3—C10	128.91 (15)	H11A—C11—H11B	109.5
C5—C4—C3	112.44 (14)	C10—C11—H11C	109.5
C5—C4—C9	121.66 (16)	H11A—C11—H11C	109.5
C3—C4—C9	125.89 (16)	H11B—C11—H11C	109.5
C4—C5—C6	129.30 (15)	C13—C12—C17	119.32 (16)
C4—C5—S1	112.85 (13)	C13—C12—N1	123.40 (16)
C6—C5—S1	117.80 (13)	C17—C12—N1	117.20 (17)
O2—C6—O3	123.37 (17)	C12—C13—C14	119.95 (18)
O2—C6—C5	126.39 (17)	C12—C13—H13A	120.0
O3—C6—C5	110.24 (14)	C14—C13—H13A	120.0
O3—C7—C8	106.38 (17)	C15—C14—C13	120.5 (2)
O3—C7—H7A	110.5	C15—C14—H14A	119.8
C8—C7—H7A	110.5	C13—C14—H14A	119.8
O3—C7—H7B	110.5	C14—C15—C16	119.61 (19)
C8—C7—H7B	110.5	C14—C15—H15A	120.2
H7A—C7—H7B	108.6	C16—C15—H15A	120.2
C7—C8—H8A	109.5	C15—C16—C17	120.50 (19)
C7—C8—H8B	109.5	C15—C16—H16A	119.7
H8A—C8—H8B	109.5	C17—C16—H16A	119.7
C7—C8—H8C	109.5	C16—C17—C12	120.15 (19)
H8A—C8—H8C	109.5	C16—C17—H17A	119.9
H8B—C8—H8C	109.5	C12—C17—H17A	119.9
C4—C9—H9A	109.5		
			
C12—N1—C2—C3	174.03 (18)	C4—C5—C6—O2	3.2 (3)
C12—N1—C2—S1	−3.6 (3)	S1—C5—C6—O2	−179.74 (17)
C5—S1—C2—N1	176.62 (16)	C4—C5—C6—O3	−176.76 (17)
C5—S1—C2—C3	−1.28 (14)	S1—C5—C6—O3	0.3 (2)
N1—C2—C3—C4	−176.94 (17)	C6—O3—C7—C8	174.54 (18)
S1—C2—C3—C4	0.91 (19)	C2—C3—C10—O1	4.0 (3)
N1—C2—C3—C10	−1.0 (3)	C4—C3—C10—O1	179.20 (18)
S1—C2—C3—C10	176.89 (13)	C2—C3—C10—C11	−175.38 (17)
C2—C3—C4—C5	0.1 (2)	C4—C3—C10—C11	−0.2 (3)
C10—C3—C4—C5	−175.39 (17)	C2—N1—C12—C13	39.8 (3)
C2—C3—C4—C9	−179.55 (16)	C2—N1—C12—C17	−143.5 (2)
C10—C3—C4—C9	4.9 (3)	C17—C12—C13—C14	−0.1 (3)
C3—C4—C5—C6	176.08 (18)	N1—C12—C13—C14	176.61 (18)
C9—C4—C5—C6	−4.2 (3)	C12—C13—C14—C15	−0.7 (3)
C3—C4—C5—S1	−1.09 (19)	C13—C14—C15—C16	0.8 (4)
C9—C4—C5—S1	178.60 (14)	C14—C15—C16—C17	−0.2 (4)
C2—S1—C5—C4	1.37 (14)	C15—C16—C17—C12	−0.6 (3)
C2—S1—C5—C6	−176.16 (15)	C13—C12—C17—C16	0.7 (3)
C7—O3—C6—O2	−2.5 (3)	N1—C12—C17—C16	−176.19 (19)
C7—O3—C6—C5	177.47 (16)		

### Hydrogen-bond geometry (Å, º)

**Table:**

*D*—H···*A*	*D*—H	H···*A*	*D*···*A*	*D*—H···*A*
N1—H1*A*···O1	0.82 (3)	1.93 (3)	2.607 (3)	140 (2)
